# Transformation of SV40-immortalized human uroepithelial cells by 3-methylcholanthrene increases IFN- and Large T Antigen-induced transcripts

**DOI:** 10.1186/1475-2867-10-4

**Published:** 2010-02-23

**Authors:** Lynn M Crosby, Tanya M Moore, Michael George, Lawrence W Yoon, Marilyn J Easton, Hong Ni, Kevin T Morgan, Anthony B DeAngelo

**Affiliations:** 1 Environmental Carcinogenesis Division, National Health Effects and Environmental Research Laboratory, US Environmental Protection Agency, Research Triangle Park, USA; 2 Curriculum in Toxicology, University of North Carolina, Chapel Hill, USA; 3 Glaxo SmithKline, Inc., Research Triangle Park, USA; 4 Sanofi-Aventis Pharmaceuticals, Inc., Bridgewater, USA; 5 Department of Physiology, University of Tennessee Health Science Center, Memphis, USA

## Abstract

**Background:**

Simian Virus 40 (SV40) immortalization followed by treatment of cells with 3-methylcholanthrene (3-MC) has been used to elicit tumors in athymic mice. 3-MC carcinogenesis has been thoroughly studied, however gene-level interactions between 3-MC and SV40 that could have produced the observed tumors have not been explored. The commercially-available human uroepithelial cell lines were either SV40-immortalized (HUC) or SV40-immortalized and then 3-MC-transformed (HUC-TC).

**Results:**

To characterize the SV40 - 3MC interaction, we compared human gene expression in these cell lines using a human cancer array and confirmed selected changes by RT-PCR. Many viral Large T Antigen (Tag) expression-related changes occurred in HUC-TC, and it is concluded that SV40 and 3-MC may act synergistically to transform cells. Changes noted in *IFP 9-27, 2'-5' OAS, IF 56, MxA *and *MxAB *were typical of those that occur in response to viral exposure and are part of the innate immune response. Because interferon is crucial to innate immune host defenses and many gene changes were interferon-related, we explored cellular growth responses to exogenous IFN-γ and found that treatment impeded growth in tumor, but not immortalized HUC on days 4 - 7. Cellular metabolism however, was inhibited in *both *cell types. We conclude that IFN-γ *metabolic *responses were functional in both cell lines, but IFN-γ *anti-proliferative *responses functioned only in tumor cells.

**Conclusions:**

Synergism of SV40 with 3-MC or other environmental carcinogens may be of concern as SV40 is now endemic in 2-5.9% of the U.S. population. In addition, SV40-immortalization is a generally-accepted method used in many research materials, but the possibility of off-target effects in studies carried out using these cells has not been considered. We hope that our work will stimulate further study of this important phenomenon.

## Background

Simian virus 40 (SV40) was first recognized and isolated during the late 1950's [1] and recently achieved fame because it was carried over inadvertently as live virus into poliovirus vaccine preparations from 1955-1963 in the U.S. and elsewhere [2]. Approximately 60% of the population in the U.S. and abroad was exposed to SV40. Initially this caused little alarm, but the virus was later found to induce mesotheliomas in hamsters [3,4] and afterwards was found in a high percentage of certain types of human cancers, especially mesotheliomas, but not in surrounding tissues [reviewed in [2]]. Discussions and investigations regarding the molecular identity of the SV40 isolates, revealed the sequences found in cancers were 'wild type', not laboratory strains [5], ruling out artifacts. Retrospective studies on human cohorts inadvertently exposed to SV40 via poliovirus vaccine increased the level of concern. A two-fold elevation in the risk of neural cancers was noted in the children of 50,000 individuals exposed to SV40 during pregnancy [5], though study design criticisms were registered. A three-fold elevation in the incidence of mesothelioma was reported in infants and children in an exposed cohort [2], and other studies reviewed therein also indicated an elevated risk of brain tumors. SV40 seroprevalence in children born in Texas from 1980-95 indicates that endemic levels of infection are 5.9% [6], or, as reviewed in Butel and Lednicky, from 3 to 13% of the number of persons not exposed to vaccine [5]. A recent investigation points to an actual prevalence of 2% [7] after correcting for cross-reactivity to JC and BK viruses. In Finland, where SV40 was not a contaminant in poliovirus vaccine, the seroprevalence is zero. A vaccine against SV40 [8] is being developed.

There is a wealth of information about the mechanisms of action of SV40 in rodent and human cells *in vitro *and *in vivo *[9-13]. SV40 Tag was found to bind and inactivate p53 and pRB, abrogating apoptotic mechanisms and control of cell proliferation, allowing cellular overgrowth and escape from senescence, and interestingly Tag-p53 complexes bind and activate the IGF-1 promoter, resulting in increased malignant cell growth [14,15]. Tag also binds the co-activators of IRF transcription, p300 and CBP [12]. Small t antigen (tag) immortalizes cells in conjunction with Tag [9], through binding and inhibition of protein phosphatase 2A. Information concerning the permissivity of human cells for SV40 has emerged [6,16] whereby p53 binding to Tag partially inactivated viral replicase activity, and then cells could support an active (but low-level) infection without a lytic component, together with a level of Tag presence which failed to activate a full-blown immune response [6]. Episomal and DNA-integrated viral replication are possible during such infection. For a more recent and extremely thorough treatment of SV40 infection, latency, and transformation of human mesothelial cells, see [17]. Testa et al. [10] suggest that asbestos and SV40 are co-carcinogenic, which may contribute to the long latency period between asbestos exposure and the development of mesothelioma (which is most often associated with the presence of SV40 virus in humans, and also with asbestos exposure). Definitive recent work by Kroczynska, et al. [18] demonstrated that crocidolite asbestos and SV40 are co-carcinogens in human mesothelial cells, and in causing mesothelioma in hamsters. In that study, SV40 did not cause malignant mesothelioma *per se*, but increased the incidence from 20% (crocidolite alone) to 90% (crocidolite + SV40).

3-MC is a well-known, thoroughly investigated, potent human and animal hepatocarcinogen of the type known as polycyclic aromatic hydrocarbons (PAH), which bind to the cytosolic Ah receptor, translocate to the nucleus via association with ARNT, and in association with ARNT bind DNA, activating transcription of genes containing XRE*'s *or ARE's [19] (such as *CYP1A1*) and eliciting an AP-1 antioxidant response. The gene expression of 3-MC has been investigated in exposed rat kidney/liver, and mouse liver [20-22] and *in vitro *in rat hepatocytes [23], and compared to other hepatotoxicants. Gene expression changes included the induction of *GSTμ, CYP1A1 *and *A2*, and several acute phase proteins in the liver, and *CYP1A1 *and *A2 *in the kidney [21]. PAH also form direct protein-DNA adducts [19].

However, the gene expression patterns induced by 3-MC in conjunction with SV40 used as an immortalizing principle have not been described. Human uroepithelial cells immortalized with SV40 (hereafter referred to as HUC) were compared to the descendant MC-SV-HUC-T-2 (hereafter referred to as HUC-TC) line which was immortalized by SV40 and subsequently transformed to tumorigenicity using 3-MC, in order to observe specific gene expression changes induced by the transforming agent. Previously, Reznikoff et al. [24] developed these cell lines and showed that treatment of HUC with SV40 followed by 3-MC, but not with either treatment individually, produced tumors in athymic mice. In the present experiment, we expected to see up-regulation of oncogenes, down-regulation of tumor suppressor genes, and other evidence of activation typical of cancer cell lines. In actuality, many were clearly virally-related when compared to the already virally-immortalized HUC, indicating a possible new interaction between viral elements and 3-MC during cellular transformation to full tumorigenicity. Here we explore those changes and discuss their possible biological significance.

## Results

### Cell Morphology and Histologic Staining

**Figure 1:**
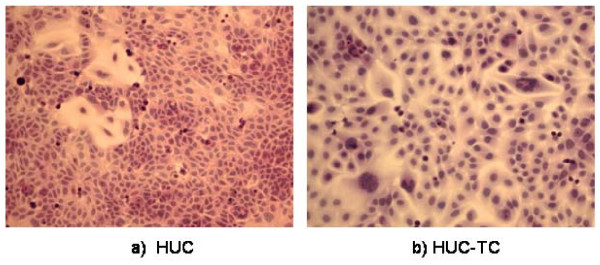
**Morphology of parent, non-transformed a) HUC and transformed, tumorigenic b) HUC-TC**. Note that HUC-TC are much larger than HUC (both a & b are same magnification) and contain giant, multinucleated cells. H & E, magnification: 20×. Bar = 50 μm.

In order to visually corroborate whether there was evidence for increased proliferation or apoptosis in either cell line, and to confirm whether HUC-TC had a more, or a less abnormal appearance than HUC (as evidenced by such criteria as heterogeneity of size or multiple/distorted nuclei) we examined HUC and HUC-TC using light microscopy. We also wished to observe first-hand whether vacuolation due to SV40 infection was present in either or both of the cell lines. We measured the proliferation of both cell lines in order to determine if a growth advantage occurred by 3-MC transformation. Untransformed, immortalized HUC appeared generally epithelioid (Fig. 1a) being rounded with faintly eosinophilic cytoplasmic staining and darker pink stippled nuclear staining. Occasionally cells displayed grossly increased cytoplasmic to nuclear ratio and numerous mitotic figures were visible. In Fig. 1b, darker staining rounded cells represent cells with condensed chromatin in prophase of the cell cycle. The cells were not contact inhibited and piled into layers and dense foci if not passaged.

**Figure 2:**
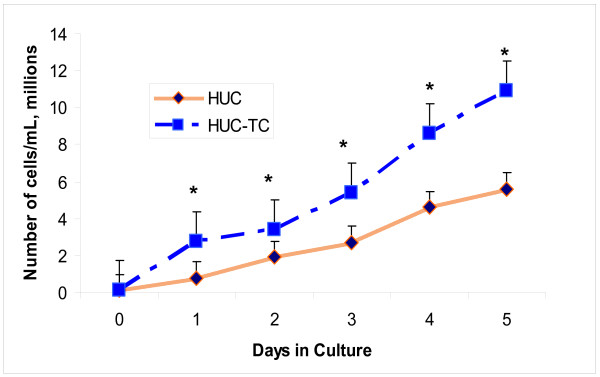
**Growth rate of HUC-TC cells was significantly higher than HUC**. (Error bars = SEM, average of 24 SD's from n = 3 experiments). Significance level = p < 0.05) for days 1-5 using two-tailed paired t-test.

HUC-TC cells (Fig. 1b) also appeared epithelioid and displayed frequent mitotic figures, but were larger than HUC. There was evidence of atypical karyotype (note cells with multi-lobulate or multiple nuclei, Fig. 1a and 1b. Vacuolation occurred in a substantial fraction of all HUC-TC cultures (Fig. 1a) as would be expected during infection with SV40. HUC-TC showed an increased tendency to form foci and grew in vertical layers vs. their non-transformed counterparts. Fig. 2 shows the growth rate of HUC vs. HUC-TC in culture under identical conditions, where it is apparent that HUC-TC possessed a significant growth advantage.

### MTS Assay for Cell Viability

**Figure 3:**
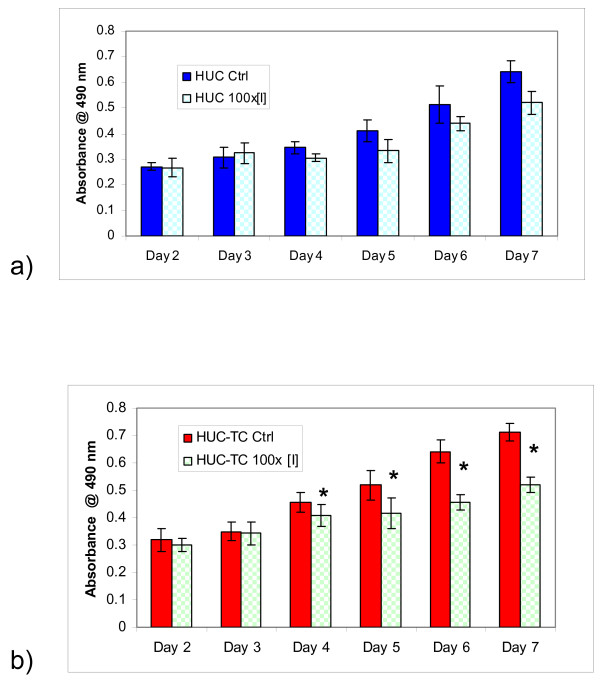
**IFN-γ suppressed metabolism in a) HUC or b) HUC-TC from days 4-7 *in vitro***. Treated (100× [I], 830 ng/mL) and control differed significantly as measured by two-tailed paired t-test for HUC-TC but not HUC. Treatment with 1×[I] concentration yielded similar results (data not shown). Error bars are ± 1 SD (n = 8).

In order to determine whether exposure of cells to IFN-γ produced cytotoxicity or reduced the cellular metabolic rate, we measured cell viability using the MTS assay after exposure to 830 ng/mL (100× [I]) of IFN-γ. From day 4 in the treatment regimen, IFN-γ suppressed cellular metabolism in a dose-dependent fashion in both cell types (Fig. 3a, b). HUC-TC growth in the presence of IFN-γ was significantly inhibited (p < 0.05, two-tailed paired t-test), however growth in HUC was not significantly inhibited using the same criteria.

### ELISA Assay for Interferon's α and γ

To explore whether the observed up-regulation of IFN-related gene expression changes (see below) could be explained, at least in part, by an increase in the secreted IFN's, levels of secreted proteins were measured. The amount of secreted IFN-γ was ~10 pg/mL, similar to that of controls in HUC and HUC-TC cell culture supernatants (data not shown). The SD between plates or wells was < 0.01. In the IFN-α assay, there was < 50 pg/mL (data not shown) (experimental dish to dish SD = 0.06, experimental well-to-well SD = 0.08) which was similar to controls.

### *In vitro *IFN-γ Treatment of Cells

**Figure 4:**
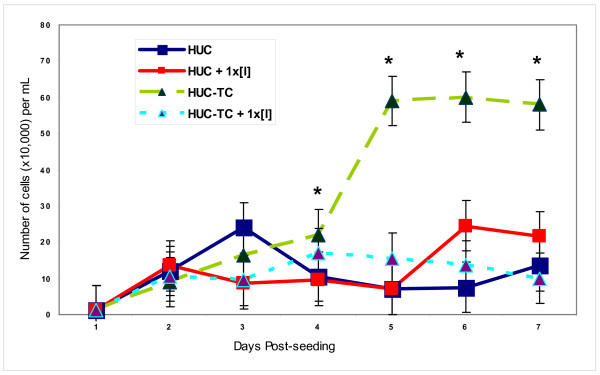
**Exogenous IFN-γ suppressed cell growth in HUC-TC (triangles and dashed lines), but not HUC (squares and solid lines) on days 4-7**. Error bars represent SEM as for Fig. 2. *p < 0.05, 2-tailed paired t-test.

In order to determine whether exogenously-supplied IFN-γ would be stimulative or suppressive of growth in transformed and non-transformed HUC if the production had been increased by transformation, we measured growth after exposing HUC and HUC-TC to inhibitory ([I]= 8.3 ng/mL) or 100× inhibitory (100× [I]= 830 ng/mL) for seven days in culture. The results of IFN-γ treatment of HUC and HUC-TC cells *in vitro *for 7 days are shown in Fig. 4. IFN-γ suppressed growth significantly (p < 0.05) only in tumor (HUC-TC) cells (triangles, dashed lines) from days four through seven. HUC treated with IFN-γ (squares, solid lines) did not show significant growth suppression.

### Gene Expression Changes

In order to better understand the cellular changes induced by transformation, differential gene expression was examined in HUC-TC compared to HUC using the Atlas^TM^ Human Cancer 1.2 Array (Cat. #7851-1). Table S1 (Additional File 1) shows the fold change in gene expression for selected gene families, with up- and down-regulation. The most clear and numerous changes represented virally related or responsive genes, many of which were interferon γ-inducible. All changes presented were significant (p < 0.05). The changes below relate to changes in HUC-TC **(**= **treated) **vs. HUC **(**= **control)**:

#### Effect of Tag on Cells (Pro-viral response)

**Figure 5:**
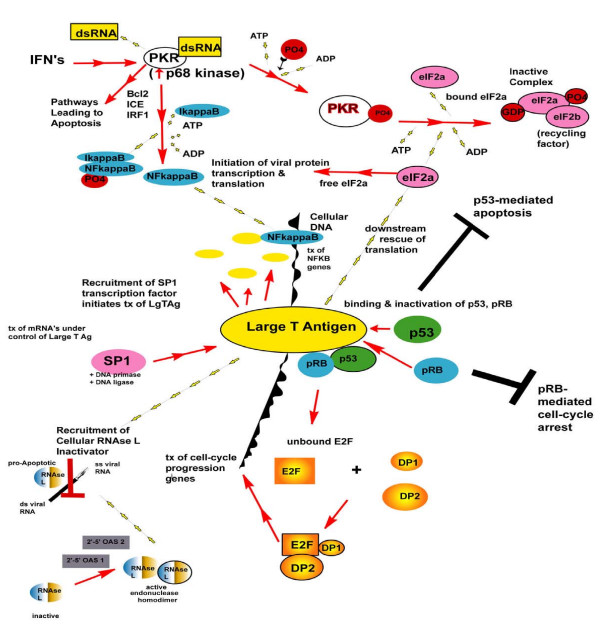
**Tag binds to and inactivates many host proteins, especially p53 and pRB**. Binding blocks pRB-mediated cell-cycle arrest and p53-mediated apoptosis. When E2F is not bound by pRB, it dimerizes with DP1 and DP2, initiating transcription of cell-cycle progression genes. PKR binds and is activated by the presence of double-stranded RNA, and activates pathways leading to apoptosis. Bound PKR activates NFκB, causing dissociation from IκB. The transcription factor NFκB binds cellular DNA and stimulates transcription of target genes resulting in either proliferation or apoptosis. Phosphorylation of PKR is initiated by binding to ds RNA, and PKR then causes eIF2a, an initiator of transcription that stimulates viral protein transcription/translation, to be released from its inactive state. Tag can also act on eIF2a causing downstream rescue of translation of viral proteins. Tag recruits SP1, a transcription factor that, together with DNA primase and ligase, initiates more Tag transcription, as well as of genes under the control of Tag. Tag also recruits RNase L-inactivator. The latter acts on ds viral RNA and converts it to inactive ss RNA, and is pro-apoptotic. The active homodimer of endonuclease RNase L is activated by 2'-5' OAS 1 & 2. See Table S1 (Additional File 1) and text for genes altered in this study.

The observed responses of HUC-TC vs. HUC that were virally-related were surprising because HUC were also SV40-exposed. Based upon extensive reviews of the function of Tag in viral infection, expected pro-viral responses include blocking antiviral responses, such as apoptosis [10-14,25,26]. See table S1 (Additional File 1) and Fig. 5 show up regulation of *TRICK2A, IAP3, HSIAH2, IRRP + DAP1 *and *TRAIL3*, which may inhibit apoptosis directly or act as decoy molecules, binding to and inactivating effectors of apoptosis. Several pro-apoptotic caspases were also up regulated, in conflict with the anti-apoptotic expression changes. Tag blocks apoptosis by binding and inactivating p53. The *Sp1 *transcription factor was up regulated 1.9-fold, and it is known that Tag recruits Sp1 in order to initiate transcription of itself and other Tag-related mRNA's, possibly by use of its DNAJ-like molecular chaperone activity. Secondly, blocking occurs through interference with PKR (RNA-dependent protein kinase, p68 kinase), which blocks the transcription and translation of viral mRNA's and proteins and is pro-apoptotic. We observed evidence of two-fold up regulated *PKR*. Thirdly, Tag blocks the action of MxA and MxAB, which also block viral mRNA transcription and protein translation. *MxA *and *MxAB *were up regulated by 8.3 and 4.6 fold, respectively, representing a response to the presence of SV40 or its components. The effects of Tag are summarized in Fig. 5.

#### Effect of IFN-γ On Cells (Anti-viral response)

Because the actions of IFN-γ are central to the innate immune response, and often occur after viral challenge, changes in IFN-γ-inducible genes were considered to be significant to SV40 exposure, but since both cell lines were SV40 exposed, this extensive response was puzzling. The IFN-γ inducible or related genes with altered expression are listed in the first section of Table S1; Additional File 1. *IFP 9-27, IFI 56, IFI 78, 1-8D + 1-8U protein*, *11.5 kDa protein, PKR*, and *IFN α/β receptor α subunit *were up regulated, among others. Down regulated genes included the *IFN-γ receptor*, which may have been either a response to increased receptor binding, the absence of ligand, or a SV40-associated thwarting of cellular mechanisms. Since no increase in secreted IFN-γ was measured, the second or third possibilities are more likely. One of the known effects of IFN-γ is an increase in the expression of MHC Class I proteins, and here several class I MHC genes were up regulated (*MHC Class truncated HLA G lymphocyte antigen, HLA DR antigen-associated invariant subunit, HLA Class I histocompatibility antigen C4α subunit*). The increase in this class of proteins is significant because cytotoxic T lymphocytes (CTLs) recognize peptide antigens in the context of class I MHC molecules, and CTL-mediated immunity is important in the defense against both cancers and viral infections [27].

#### Signal Transduction

**Figure 6:**
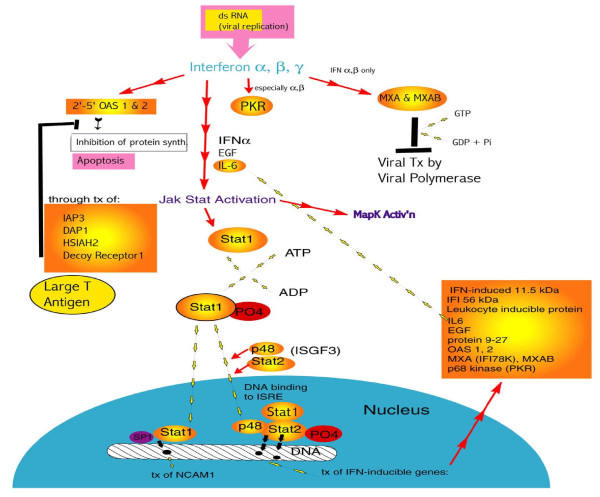
**How the presence of virus acts on innate immune system components, i.e. interferons, activating the Jak-Stat pathway**. Double-stranded RNA, the product of viral replication, is recognized by interferons α, β, and γ and they in turn (especially α and β) activate PKR. See Fig. 5 for PKR actions. Interferons also activate MXA and MXAB which block transcription of viral products by the viral polymerase, and activate 2'-5' OAS 1 & 2, which inhibit viral protein synthesis and stimulate apoptosis. However Tag may block the actions of 2'-5'OAS 1 & 2 by initiating transcription of inhibitors of apoptosis genes (see small orange sunburst block). Interferon α, EGF, and IL-6 activate Jak-Stat pathways, activating Stat1 which becomes phosphorylated and binds p48 and Stat2 (the trimer is ISGF3 or STAT1), bind to the ISRE on DNA and initiate transcription of IFN-inducible genes (one of which is IL-6, see large orange sunburst box). Stat1 also binds SP1 and this dimer initiates transcription of NCAM1, a cell adhesion molecule. Genes colored in yellow-orange denote altered gene expression levels measured.

Typically, during transformation cell signalling becomes altered. Aspects of MAPK, Ras, Sonic Hedgehog and Jak-Stat signaling were affected, some of which were explained by known responses to viruses. The Jak-Stat response to interferons (Fig. 6) shows gene expression changes observed in agreement with the literature. Jak-Stat activation occurs with the ATP phosphorylation of Stat-1, followed by its translocation to the nucleus, where it may dimerize with SP1 (up regulated here) and initiate the transcription of *NCAM1*, or form a trimer with p48 and Stat-2 (up regulated here) and initiate the transcription of IFN-inducible genes (see above and Fig. 6). The down-regulation of the *IL-6 *precursor may represent a negative feedback loop for Jak-Stat pathway de-activation.

#### Extracellular Matrix Reorganization

Because the ECM is typically degraded during cancerous cell invasion, these changes may have been related to 3-MC exposure. All ECM gene expression changes were decreases (see Table S1, Additional File 1). The changes within this group included *collagens, cytokeratins, integrins and glucocorticoid receptors *which may be related to the induction of metastasis. Several matrix metalloproteinases and N-gal (neutrophil gelatinase), which are associated with tumor invasiveness, were up regulated.

#### DNA Damage Response

**Figure 7:**
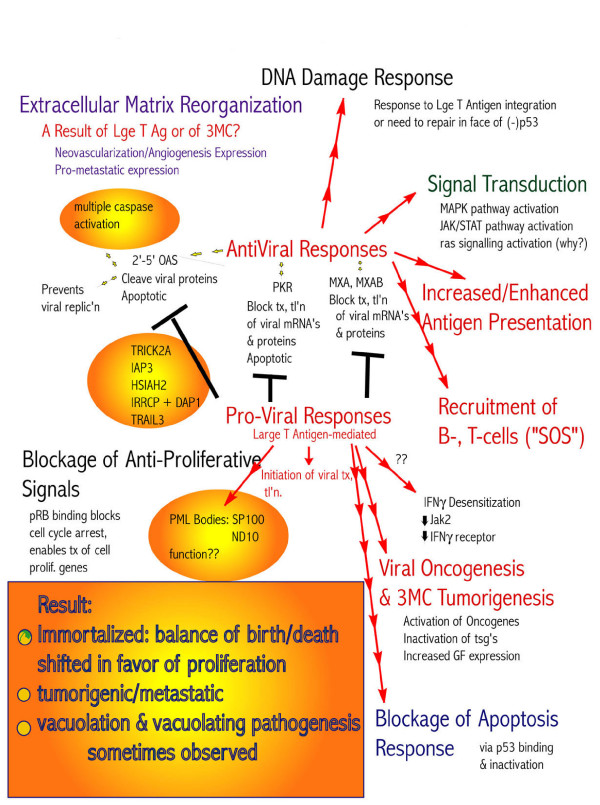
**3-MC treatment of SV40-immortalized cells induced pro- (mediated by Tag) and anti- (mediated by innate immune system) viral responses, extracellular matrix reorganization, antigen presentation, recruitment of B- and T-cells, activation of MAPK, JAK/STAT, ras, etc**. signal pathways, blockage of apoptotic response, and stimulation of proliferation (through blockage of cell cycle arrest), together with activation of oncogenes and inactivation of tumor suppressor genes (tsg's), as well as a DNA damage response (with possibly defective repair). This resulted in a shift of the balance of birth and death toward proliferation, development of a tumorigenic/metastatic phenotype, and sometimes, the vacuoles typical of SV40 viral infection.

DNA damage might have been caused by exposure to 3-MC, and could have been defective, resulting in transformation to carcinogenicity. Several DNA damage response genes showed altered expression, most notably *GADD 153 *(4.1 fold up regulated). *XPG group E, XPG DNA excision repair, DNA mismatch repair PMS1, DNA recombination & repair protein HNGS1 *were up regulated (see Table S1, Additional File 1). Down regulated genes included *DNA Ligase IV, ERCC1 and XPD group D*. The gene expression results are summarized in Fig. 7 for pro- and anti-viral responses and their end results, showing how these changes might be related to transformation.

### TaqMan™ Quantitative RT-PCR Confirmation of Selected Gene Changes

**Figure 8:**
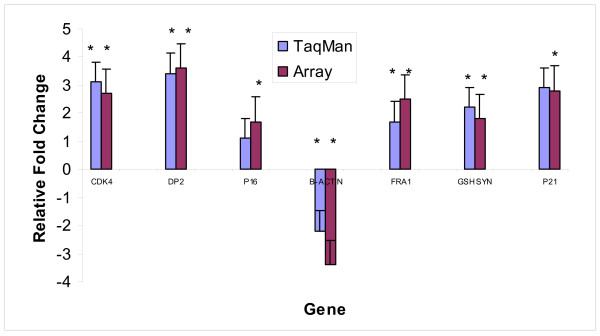
Comparison of RT-PCR and array data showing good agreement between methods for 7 selected genes (β-actin = endogenous control) in HUC-TC vs. HUC cells (*significantly different than HUC at p < 0.05). Error bars = SEM.

Several genes were chosen to corroborate the gene expression results obtained from the arrays. The genes *CDK4, DP2, p16, β-actin, FRA1, GSH synthetase and p21*^*waf1*/*cip*1 ^were chosen based on relevance to the mechanisms of action of SV40 (and in the case of GSH synthetase, 3-MC) and strong response on the gene expression array. Fig. 8 shows the relative fold-change in expression using the Taqman™ assay (n = 5), where all changes except p16 were significant at the level of p < 0.05, and the Clontech™ gene expression array, where all changes measured were significant at p < 0.05. The intra-sample variance was 0.05, 0.06 and 0.10 for *cdk4, dp2 and p16ink4*, respectively, e.g., and the maximum fold change was 1.5. Close agreement was achieved between the two methods.

## Discussion

The morphology, growth characteristics, phenotype, karyotype, and ultrastructure of these cell lines were extensively described previously [24,28-35]. The parent HUC non-transformed cell line did not produce tumors after inoculation *in vivo *up through at least passage 80 (over 3 years) in culture. However, the parent cell line was highly unstable chromosomally. Wu et al. [33] demonstrated that marker chromosomes of 3 tumor cell lines (HUC-TC) were stabilized relative to the parent non-transformed cell line, by malignant transformation. HUC-TC were transformed at passages 12-15, and we obtained cells from the repository that were passage 14. We used these cells at passage 19. We obtained the parent HUC non-transformed cell line at passage 32 and used it at passage 38. We inoculated these HUC-TC (passage 19) into athymic mice and tumors were produced in the same manner as the original experiments [24], (data not shown). Given the previous extensive characterization of these cells and the limited number of passages that elapsed between the time we obtained and used the cells for experimentation, the likelihood of significant alterations in the genome is limited, but cannot be completely ruled out.

It was expected that the gene expression results would strongly reflect the 3-MC treatment. We chose to use the human cancer array and therefore changes in other metabolic genes such as *CYP1A1*, which is also known to occur upon 3-MC treatment, were not measured. The gene expression changes seen upon comparing HUC with HUC-TC were surprising in that they were highly related to SV40 treatment although both cell types had been SV40-treated. It appeared that a non-transient (i.e., present through several cell passages) expression and enhancement of anti-viral responses occurred in HUC-TC as a result of the treatment with 3-MC. Below we discuss how this activity might result in carcinogenesis.

Cellular antiviral responses typically begin with host cell recognition of the internal presence of SV40 double-stranded RNA, an indicator of viral replication [25]. The response includes up-regulation of *IFN's α/β/γ*, with multiple effects such as up regulation of the expression of *2',5'-OAS 1 and 2*, seen here, activating the RNase L homodimer. Active RNase L then cleaves double-stranded viral RNA and stimulates apoptosis. But clearly apoptosis was not activated (Fig. 1, which is a representative image, shows no apoptotic cells or bodies and Fig. 2 shows that both cell types had a healthy growth rate in culture). The activation of PKR by type I interferons would then typically result in binding of eIF2a to GDP and eIF2b, a recycling factor for eIF2a, inactivating eIF2a and blocking the initiation of protein translation. (The down-regulation of the related *eIF4E 25-kDa subunit*, in this experiment, may have been another mechanism of decreased protein translation.) PKR then typically activates NFκB, which translocates to the nucleus, binds DNA in the promoter regions of NFκB-responsive genes, and initiates transcription of proliferation-related or stress-responsive genes, the latter of which lead to apoptosis. PKR activation blocks viral transcription and translation, as does the up regulation of *MxA *and *MxAB *(up regulated here) in response to interferons. Here, PKR may have stimulated pro-proliferative genes but pro-apoptotic genes may have been incompletely or improperly activated, or such activation may have been ineffective due to the up regulation of opposing signals (see below). Waring, et al. [20] have identified a gene expression profile that is similar to that of 3-MC and mediates hepatic toxicity through the AhR either directly or through the effects on NFκB, resulting in the inhibition of cell adhesion protein expression. If such a pathway acts through NFκB, it may be similar to the PKR-mediated NFκB activation pattern seen here, producing a tumorigenic phenotype. Additional pro-apoptotic elements were up regulated (*endonuclease III, caspase 4, caspase 10, death receptor 5 (TRICK2A), TNFRSF25 (WSL + TRAMP + Apo-3 + DDR3)*) however these cells were not apoptotic. The reason for unchecked proliferation may be related to the up regulation of multiple blockers of apoptosis (*DR1, HSIAH2, IAP3, DAP1*), known to act either as decoys that bind and inactivate apoptotic ligands, or act upstream of the caspases. In addition, pRB is known to be bound by Tag, nullifying cell cycle checkpoint control. p53 protein was at least partly functional in these cells, as we noted several *p53-inducible gene *expression increases, as well as *mdm2 *up regulation. However Tag is known to bind p53 and render it incapable of initiating apoptosis. Although p53 and pRB binding by Tag can account for both loss of apoptosis signaling and checkpoint control, there were many other changes at the mRNA level related to these important functions and indicative of cellular dysregulation.

Cell-cycle arrest was signaled as well, since *p21*^*waf*1/*cip*1 ^(up regulated) is a p53-inducible universal CDK inhibitor and its up regulation is known to inhibit cell proliferation. The response was clearly not successful, most likely due to pRB-Tag binding. Tag was present in these cell lines [32], and there was evidence of an increase in the rate of proliferation in HUC-TC vs. HUC. Other cell-cycle genes up regulated include *CDK4/cyclin D2 *and *CDK7*. CDK7 together with cyclin H forms CAK, a kinase required for CDK activation. Although *p16*^*ink*4 ^was up regulated, it could not bind pRB, which would have been already bound by Tag, and so could not block cell cycle progression. Ultimately, apoptosis was blocked and cell-cycle control circumvented (Fig. 5, 6, 7).

These results imply stimulation of IFN-γ-related pathways by 3-MC. Treatment with exogenous IFN-γ blocked cell proliferation in tumor, but not non-tumor HUC. However metabolic activity (as assayed by MTS) was decreased in both cell lines treated with IFN-γ from day 4 onward. Since there was no elevation in the level of secreted IFN α or γ, and many IFN-γ-inducible transcripts were increased, we conclude that 3-MC treatment activated IFN pathways without affecting constitutive levels of IFN. An hypothesis is that activation of IFN-γ-related pathways by 3-MC rendered HUC-TC susceptible to growth-suppression by exogenous IFN-γ. These data support the idea that during immortalization (first step, SV40-mediated) cells become unresponsive to IFNγ-mechanisms of cell cycle control, but subsequently, during transformation (second step, 3-MC-mediated) cells are altered in such a way that they are rendered sensitive to IFNγ control of cell proliferation, but by then it is too late because other aspects of cellular function controlling growth have been irrevocably altered. The cell cannot 'retreat' along the pathway to which it has become immutably committed, i.e. immortality. The 'coup de grace', 3-MC transformation of the primed cell population, might then be facile.

Clearly the IFN-γ pathways activated by 3-MC were not *intrinsically *growth-suppressive in nature, since HUC-TC exhibited more rapid growth than HUC in the absence of treatment with exogenous IFN-γ. Activation of IFN-γ-inducible gene expression may represent dysregulation of homeostatic IFN-γ pathways. This raises the question of how the altered pathways promote tumor growth and metastasis. We would remind the reader that it is known that a slight deviation in one or more components of a growth-suppressive pathway may alter the function of the entire pathway, achieving the opposite effect, e.g. TGFβ signalling either promoting or suppressing tumors [36]. Demonstration of the suppressive effects of IFN-γ on cancer cell growth both *in vitro *and *in vivo *has been unequivocal [37-39] and the production of IFN-γ in response to chemotherapy is one marker used to assess the success or failure of treatment *in vivo*; it is considered an indicator of immune activation and anti-tumor activity [40,41]. In addition, studies of infectious diseases (scrapie, herpes simplex virus type 1, human cytomegalovirus, human influenza virus C, type 1 diabetes) have linked IFN-γ inducible gene expression with the presence of disease and/or anti-viral mechanisms [42-45]. In a recent study employing HTLV-1 to transform Rat-1 fibroblast cells *in vitro*, four of eight up regulated genes were IFN-stimulated genes, and the 2',5'-OAS promoter was activated by viral Tax indirectly through an NFκB-dependent pathway, linking IFN-signaling with Tax transformation [46]. We observed evidence of PKR up-regulation, which can directly activate the NFκ-B pathway. The present study may be another example of transformation that occurs via IFN-γ pathways.

The most highly up regulated gene was *lipocalin *(*neutrophil gelatinase-associated lipocalin, NGAL*). Lipocalin has been found in a high molecular weight complex associated with progelatinase B, one of several gelatinase isoforms commonly found in the urine and cancerous tissues of bladder cancer patients [47]. Since 3-MC is a known human uroepithelial carcinogen which has previously tested positive for tumorigenicity in mice *in vivo *[48], this transcriptional up regulation appears to be a correlate for urothelial malignancy, and makes *lipocalin *a biomarker of exposure as others have observed for bladder cancer [49], as well as colorectal cancer [50], and as reviewed in [51].

These data provide evidence that SV40 and 3-MC may act synergistically to promote transformation to a tumorigenic phenotype. Inoculated HUC-TC produced tumors in athymic mice as in the original experiments and we found gene expression changes related to viral elements to be up regulated, along with many interferon-responsive genes. We hypothesize that first SV40 infection altered cellular pathways related to cell cycle control and apoptosis, then 3-MC exposure initiated changes in interferon response elements, matrix attachment proteins, DNA damage responses, and activation of oncogenes and/or inactivation of tsg's, resulting in transformation to the malignant phenotype. This may be relevant to human environmental exposures.

## Methods

### Cell Culture, Morphology and Histologic Staining

HUC and HUC-TC were obtained from The American Type Culture Collection, Rockville, MD at passage 32 (HUC) and 14 (HUC-TC). HUC (passage 38) and HUC-TC (passage 19) cells were plated onto 150 mm dishes (five per treatment) at a density of 1 × 10^5 ^cells/mL and allowed to reach 80 - 90% confluence, or 5 days, under standard culture conditions. Cells were fed three times per week (26 mL/dish MEM containing 10% FBS, 0.1 mM non-essential amino acids, 2 mM L-glutamine, 2.7 mg/mL D-glucose, 10 μg/mL insulin, 5 μg/mL transferrin, 0.1 μg/mL hydrocortisone). Glass coverslips were placed into the 150 mm dishes at the time of plating and carefully removed with forceps before RNA harvest was carried out on the remaining cells. Cells from glass coverslips were fixed by rinsing in room temperature sterile PBS and fixed in cold (-20°C) absolute ethanol for 24 h, stained using Hematoxylin and Eosin by standard histologic staining methods, and photographed.

### MTS Assay for Cell Viability

The Promega Cell Titer 96 Aqueous One Solution™ assay was employed to measure the metabolic activity of IFN-γ-treated HUC and HUC-TC cells relative to control cells. This assay relies on the conversion of a tetrazolium compound (MTS) to a blue colored reduced formazan product, which requires cellular reducing capacity as NADH and NADPH. Cells that are not metabolically competent will not reduce MTS. Cells (HUC = passage 37, HUC-TC = passage 18) were plated at a density of 1.25 × 10^4 ^cells/mL into 96-well plates (Corning plastic, one plate/cell type/day for a total of 12 plates) and grown for 7 days. Cells were fed with fresh media, 1× [I] (where [I] = 8.3 ng/mL) or 100× [I], IFN-γ on days 2, 4 and 6. On days 2 - 7 one plate of each cell type was assayed using the MTS reagent. 20 μL of MTS reagent was added to each well and plates were incubated in the dark under standard tissue culture conditions for one hour. Optical density was measured on a Titertek Multiskan™ spectrophotometer at 490 nm. 8 wells were read per treatment condition, on each plate, and the readings averaged. Statistical analysis was carried out using an Excel™ spreadsheet and significance levels analyzed using a paired two-tailed t-test.

### ELISA Assay for Interferon α and γ

Assays for quantitation of secreted interferons α and γ were performed in a 96-well format using commercially obtained assay kits. A Quantikine (DIF50, R & D Systems, Mpls., MN) kit was used for human IFN-γ including calibrated pure recombinant human interferon standards and a polyclonal antibody specific for human IFN-γ. A similar IFN-α kit was obtained from PBL Biomedical Laboratories, Inc. (product #41400, v.2.2). Standard curves for each were constructed and interferons were quantitated in pg/mL, according to manufacturers' instructions. HUC-TC cells (passage 38 or 39 for IFN-α or IFN-γ respectively) were plated at a density of 1.25 × 10^4 ^cells per mL into six dishes per cell type, and 100 μL of purified cellular supernatant per well was pipetted into the antibody-coated 96-well plate. The assay was carried out per the manufacturer's instructions, and results were read spectrophotometrically. Statistical analysis was carried out using an Excel™ spreadsheet.

### *In vitro *IFN-γ Treatment of Cells

To assess the effect of IFN-γ on cell growth in culture, HUC (passage 40) and HUC-TC (passage 21) were treated with a known inhibitory concentration [I] of 8.3 ng/mL recombinant human IFN-γ (specific activity 2 × 10^8 ^U/mg, #I-3265, Sigma Chemical, St. Louis, MO) or control media 1 day post-plating, and grown for six days without media replacement. On day zero, cells were plated into 24 each 25 cm^2 ^flasks at a density of 1.25 × 10^4 ^cells/mL (six control and six treated for HUC and HUC-TC). One dish from each treated and control dish was trypsinized using standard methods and counted each day beginning on day two post-plating. Counts were taken using a standard hemacytometer, in duplicate, and the results averaged. Significance was determined using an Excel™ spreadsheet and a paired two-tailed t-test.

### RNA Preparation and Labeling of cDNA and Hybridization to Arrays

RNA was extracted by the addition of 14 mL TRIZOL™ reagent (Gibco BRL, Grand Island, NY) after triple rinsing with sterile room temperature PBS, according to the manufacturer's protocol. Six μg of total RNA per sample was reverse-transcribed and radioactively labeled using α^33^P-dCTP in a previously-described PCR reaction [52]. Labeled cDNA was hybridized overnight at 64°C and washed free of unhybridized cDNA in 0.5× SSC/1% SDS once, then twice in 2× SSC/1% SDS (20 min. per wash) at 64°C. Membranes (Atlas™ Human Cancer 1.2 Array [Cat. #7851-1], n = 5 per treatment) were exposed for 48 h to a rare-earth screen and read on a phosphorimager (Cyclone™ Scanner).

### Data Manipulation & Statistical Analysis

The resulting intensities were uploaded into the Atlas Image 1.5™ software program (Clontech Inc., Palo Alto CA). Membranes were then aligned according to the manufacturer's instructions using the global normalization option and screened for bleed or other anomalies. The resulting reports were analyzed by group, for statistical significance (p < 0.05), using the NoSeCoLoR (NLR) software program, a normalization and local regression program [53] as in previous studies [52]. Statistically significant results were interpreted by use of current literature and diagrams constructed integrating experimental results with known biological pathways.

### TaqMan™ Quantitative RT-PCR Confirmation of Selected Gene Changes

Using RNA from the same experiment as for gene expression, the expression changes of selected strong responding genes were confirmed using a Taqman™ real time quantitative RT-PCR assay (Perkin Elmer Prism ABI), as previously published [52]. Primers were designed using Perkin Elmer Primer Express™, purchased from Keystone Biosource Inc. (Camarillo, CA) and prepared according to the manufacturer's instructions. The genes chosen for this assay were: *CDK4, DP2, p16*^*ink*4^, *β-actin *(endogenous control), *FRA-1, GSH synthetase and p21*^*waf*1/*cip*1^. These genes were altered on the array at p < 0.05, and were relevant to the mechanism of action, as observed by array results. The ΔΔCT method was used to calculate the fold-change in gene expression for the selected genes. β-actin was used as the endogenous control.

## List of abbreviations

3-MC: 3-methylcholanthrene; SV40: Simian virus 40; HUC: human uroepithelial cells; HUC-TC-human uroepithelial cells (tumor cells); IFP 9-27 'protein 9-27': interferon-inducible protein 9-27; IF 56: interferon-inducible 56 kDa protein; MxA: interferon-regulated resistance GTP-binding protein (IFI-78K); MxAB: interferon-regulated resistance GTP-binding protein; IFN-γ α, β: interferon gamma; alpha; beta; Tag: Large T antigen of SV40; tag: small t antigen of SV40; p53: tumor suppressor protein p53; pRB: retinoblastoma protein; CBP: CREB binding protein; transcriptional co-activator for IRF genes; p300: as a dimer binding to CBP; forms a transcriptional co-activator for IRF genes; Ah receptor: aryl hydrocarbon receptor; ARNT: Ah receptor nuclear translocator; XRE: xenobiotic response element; ARE: antioxidant response element; CYP1A1: cytochrome p450 1A1; AP-1: jun/fos dimer; activator of transcription; α^33^P-dCTP: the α-labeled; phosphorus 33 radioisomer of deoxycytidine triphosphate; CDK4: cyclin-dependent kinase 4; DP1 and 2: dimerization partner 1 and 2; p16^ink4^: tumor suppressor protein p16 mRNA; β-actin: "housekeeping" gene; cytoskeletal-associated; Fra-1: fos-related antigen 1; GSH synthetase: glutathione synthetase; p21^waf1/cip1^: universal cyclin-dependent kinase inhibitor; TRICK2A: death receptor 5 or DR5 (also Apo2; TRAIL-R2; TRICK2; or KILLER); IAP3: inhibitor of apoptosis protein 3; HSIAH2: inhibitor of apoptosis protein 2; IRRP + DAP1: death-associated protein + death-associated protein 1; TRAIL3: TNF-related apoptosis-inducing ligand 3; Sp1: transcription factor; PKR: RNA-dependent protein kinase; p68 kinase; DNA-J: a molecular chaperone; 1-8D + 1-8U protein: interferon inducible protein mRNA; MHC: major histocompatibility complex; HLA: human lymphocyte-associated; DR: decoy receptor; NCAM1: neural cell adhesion molecule 1; JAK-STAT: Janus kinase and signal transducer and activator of transcription; p48: damage specific DNA binding protein subunit mRNA; IL-6: interleukin 6 mRNA; ECM: extracellular membrane; GADD 153: growth and DNA damage protein 153; XPG: xeroderma pigmentosum; PMS1: mismatch repair gene; HNGS1: DNA recombination and repair protein mRNA; ERCC1: DNA excision repair gene; 2',5'-OAS: 2',5' oligoadenylate synthetase; eIF2a: eukaryotic translation initiation factor 2 alpha; GDP: guanidine diphosphate; NFκB: nuclear factor kappa B; mdm2: feedback regulator of p53 (an ubiquitin ligase); CAK: cyclin-associated kinase (activator of CDK's); NGAL: lipocalin (neutrophil gelatinase-associated lipocalin precursor); E2F: transcription factor; ICE: interleukin-converting enzyme, ISRE: interferon-stimulated response element; ISGF3: STAT1; tsg's: tumor suppressor genes; tl'n: translation; tx: transcription; WSL+TRAMP+Apo-3+DDR3: TNFRSF 25 (TNFRSF12).

## Competing interests

The authors declare that they have no competing interests.

## Authors' contributions

LMC designed the study and carried out the *in vitro *experiments, analyzed the data and wrote the manuscript. TMM and MG carried out animal studies that were the basis for this study. LWY and HN performed Taq Man^® ^real time RT-PCR and gene expression array experiments, respectively. KTM and ABD participated in planning the studies and analysis and interpretation of the data. All authors have read and approved the final manuscript.

## Supplementary Material

Additional file 1 **Table S1** - Gene Expression Changes in 3-methylcholanthrene transformed HUC (p < 0.05). Click here for file
