# Correction: Transformation of SV40-immortalized human uroepithelial cells by 3-methylcholanthrene increases IFN- and Large T Antigen-induced transcripts

**DOI:** 10.1186/1475-2867-10-10

**Published:** 2010-04-14

**Authors:** Lynn M Crosby, Lawrence W Yoon, Marilyn J Easton, Hong Ni, Kevin T Morgan

**Affiliations:** 1 Department of Physiology, University of Tennessee Health Science Center, Memphis, USA; 2 Glaxo SmithKline, Inc., Research Triangle Park, USA; 3 Sanofi-Aventis Pharmaceuticals, Inc., Bridgewater, USA

## 

After publication of this work [1], US Environmental Protection Agency (EPA) requested the removal of US EPA authors due to lack of clearance. This manuscript was not reviewed or approved by the agency, nor is anything in it deemed to be recommended by, or in accordance with US EPA views.

## Author Agreement

All authors have been notified of, and agree with this decision.

## Competing interests

The authors declare that they have no competing interests.

## Authors' contributions

LMC designed the study and carried out the *in vitro *experiments, analyzed the data and wrote the manuscript. LWY and HN performed Taq Man^® ^real time RT-PCR and gene expression array experiments, respectively. MJE assisted with *in vitro *experiments. KTM participated in planning the studies and analysis and interpretation of the data. All authors have read and approved the final manuscript.
