# Supplementary material for: Transformation of SV40-immortalized human uroepithelial cells by 3-methylcholanthrene increases IFN- and Large T Antigen-induced transcripts
Source: Cancer Cell Int. 2010 Feb 23;10:4. doi: 10.1186/1475-2867-10-4 (PMC2848030; doi:10.1186/1475-2867-10-4)
Supplement: Additional file 1 — Table S1 - Gene Expression Changes in 3-methylcholanthrene transformed HUC (p < 0.05). [file 1475-2867-10-4-S1.PDF]

Table I.
Gene Expression Changes in 3-methylcholanthrene transformed HUC ($p < 0.05$)

blue= down-regulated expression relative to non-transformed
yellow = up-regulated expression relative to non-transformed

Anti-viral and interferon-related genes:

Gene I.D.	Fold-change	Gene I.D.	Fold-change
Interferon-inducible protein 9-27	13.6	CD82 antigen (metastasis suppressor)	3.2
2'-5' oligoadenylate synthetase 1	12.4	JAK2	2.3
2'-5' oligoadenylate synthetase 2	10.1	DAP2 (DAPK1)	2.2
Interferon-inducible 56 kDa protein	9.9	IL-6 precursor	2.2
Leukocyte interferon-inducible peptide	9.7	MIF (macrophage migration inhibition factor)	2.1
MXA interferon-regulated resistance GTP-binding protein (IFI-78K)	8.3	PI-3 kinase	1.9
MXAB Interferon-regulated resistance GTP-binding protein	4.6	Interferon γ Receptor (CDW 119 antigen)	1.8
HLA DR antigen-associated invariant subunit	3.4	Activator 1 40 kDa subunit; replication factor C 40 kDa subunit (RFC 40)	1.8
CD27 ligand: CD70 antigen	3.2	FC epsilon receptor gamma subunit	1.7
Interferon-inducible protein 1-8D + 1-8U	3.1		
LMP2 MHC-encoded proteasome subunit	3		
STAT1	2.7		
DNA primase small subunit (49 kDa)	2.6		
Interferon α-induced 11.5 kDa protein	2.5		
HLA Class I histocompatibility antigen C4α subunit	2.3		
Lymphotoxin β receptor precursor (TNF receptor 2-related)	2.2		
IL-1α precursor	2.1		
IgG receptor FC large subunit p51	2.1		
MHC Class 1 truncated HLA G lymphocyte antigen	2.1		
Interferon-inducible RNA-dependent protein kinase (P68 kinase) (PKR)	2		
Interferon α/β receptor α subunit precursor	2		
IL-6 receptor α	1.9		
SP-100 protein, lymphoid restricted homolog	1.9		
SP1 transcription factor	1.9		
Nuclear domain 10 protein 52, associated with SP-100, PML bodies	1.7		
STAT5A	1.7		
Oncostatin M-specific receptor β subunit (IL-6 death associated protein)	1.7		

Viral Replication:

DNA primase small subunit (49 kDa)	2.6	DNA ligase IV	2.1
		Replication factor C	1.8

Retinoblastoma & p53-associated Genes:

PAKα	5.4	MDMX mdm2-like	3.8
MDM2	5.3	RB1 (p105)	1.9
DP2	3.6		
RBP2	2.3		
RBP4 chromatin assembly factor 1 p48	2		
53BP1	1.8		
PIG7	1.7		
p16 ink4	1.7		
DP1	1.6		

Oncogenes/Tumor suppressor genes:

NGAL (lipocalin, neutrophil gelatinase-associated lipocalin precursor	29.2	MDMX	3.8
MDM2	5.3	CD82 antigen (metastasis suppressor)	3.2
VEGF	4.2	ABL2	3.2
MCC	2.7	c-jun	2.7
Fra-1	2.5	ETS-1	2.1
NOTCH1	2	ELF1	2.1
c-Myb	1.9	VEGFB	2
ATL-derived PMA-responsive peptide APR	1.9	RB1 (p105)	1.9
53BP1	1.8	Leukemia-associated gene 1	1.9
Damage-specific DNA binding protein p48 subunit (XRCC E)	1.7	Cullin homolog 2 (CUL2)	1.8
Polycystin precursor	1.7	Shb proto-oncogene	1.6
Oncofetal antigen precursor 5T4	1.7	SMAD4 (DPC)	1.6
B-raf (RAFB1)	1.7	ODC	1.8
IRRP (DAP)	1.7		
c-Myc	1.6		
MCL-1	1.6		

Apoptosis-associated Genes:

TRAIL receptor 3 (decoy receptor 1)	4	DAPK1	2.2
HSIAH2 (IAP2)	2.8	Survivin apoptosis inhibitor	2.2
Endonuclease III homolog 1	2.6	HIAP2 (IAP2 + IAP homolog B) TNFR2 related	1.8
Caspase 4 precursor (ICH-2 protease)	2.1		
Caspase 10 precursor	2.1		
IAP3	2		
TRICK2A (DR5)	2		
WSL + TRAMP + Apo3 + DDR3	1.8		
IRRP (DAP)	1.7		
Caspase 9 (ICE-LAP6)	1.7		

Cell Cycle Control-Related Genes:

cyclin D2 (G1/S)	5.7	mdm2-like MDMX	3.8
PAKα	5.4	ARK1	2.7
mdm2	5.3	protein D123	2.3
ribosomal protein S6 kinase	3.5	cyclin-dependent kinase regulatory subunit CKS2	2.3
p21/waf1/cip1	2.8	CHK1	2.3
CDK4	2.7	CDK5	2.2
SAPK4	2.6	cyclin B1	2
CDK2	2.3	cdc25A	1.9
p38-2G4 homolog HG4-1	2	CDK6	1.8
cdc2-related kinase PISSLRE	1.8	cyclin D1 (G1/S)	1.7
53BP1	1.8	cyclin A (G2/M)	1.6
CDK7	1.8	cdc2 p34 kinase	1.6
PIG7	1.7		
p16ink4	1.7		

ERK/MAPK Pathway-Related Genes:

MAPKK5	2	ERK4 (MAPK4)	14.4
JNKK2	1.7	ERK5	1.8
RafB1	1.7		

Ras Signaling Pathway-Related Genes:

MXA GTP binding protein	8.3	Rho GDP dissociation inhibitor (RHO GDIβ)	8.3
MXAB GTP binding protein	4.6	Caveolin 2	2.8
ras-related RAP-1A	2.3	Transforming protein Rho A (Rho-12)	1.9
Cyclic-AMP dependent transcription factor ATR-1 (TREB36 protein)	2	cAMP response element modulator 1 alpha protein HCREM	1.8
RafB1	1.7	Caveolin 1	1.8
		Rho-related GTP-binding protein RHO E (Rho 8)	1.8
		GRB2-associated binder 1 (GAB1)	1.7
		Ras related C-botulinum toxin substrate p21 rac-1 ras like protein	1.6
		Rho C small GTPase	1.6

Viral Attachment/Antigen Presentation-Related Genes:

Myeloid plasma membrane glycoprotein CD13	8.5	Vimentin	6.5
Microtubule-associated protein 1B	4.5	Fibronectin	5.5
Fibronectin receptor α subunit (integrin α5)	2.6	Cadherin 2 (neural cadherin)	4.9
Metalloproteinase inhibitor 3 precursor (TIMP 3)	2.5	Integrin β1	4
Matrix metalloproteinase 14 precursor	2.3	Cytoplasmic β actin	3.4
Collagen 6α1 subunit	2.3	N-CAM L1	3.4
HLAC HLA class I histocompatibility antigen C-4α subunit	2.3	CD82 antigen metastasis suppressor	3.2
Lymphotoxin β receotir precursor	2.2	Cadherin 5 (VE-cadherin)	3
Matrix metalloproteinase 15	2.2	Collagen 8α subunit	2.8
Cadherin 3	2.1		
IgG receptor FC large subunit P51	2.1		
HLAG MHC Class I truncated HLA G lymphocyte antigen	2.1		
Platelet membrane glycoprotein IIIA precursor (integrin β3)	2		
Mesothelin	1.9		
B-cell receptor-associated protein	1.8		
Integrin α1; laminin & collagen receptor	1.8		
Cytokeratin 14	1.7		
Importin β1 subunit	1.7		

Metalloproteinase Genes:

VEGF	4.2	Vimentin	6.5
TIMP3	2.5	Fibronectin	5.5
MMP14	2.3	CD82 metastasis suppressor	3.2
MMP15	2.2	MT-III	2.6
TGFβ	2	Thrombospondin 2 precursor	2.1
		Vitronectin	2.3

DNA Repair/Damage Sensitive Genes:

GADD 153	4.1	XPD Gp D (DNA Excision repair)	2.2
DNA primase small subunit 49 kDA subunit	2.6	DNA Ligase IV	2.1
DNA mismatch repair PMS1	2	ERCC1 (DNA Excision repair)	1.7
XPG DNA excision repair (xeroderma pigmentosum)	1.8		
DNA recombination & repair protein HNGS1	1.8		
Damage specific DNA binding protein p48 subunit (DDBB p48), XPG group E	1.7		

Sonic Hedgehog Signaling Pathway-Related Genes:

Smoothened	2.4	Cadherin 2	4.9
Cadherin 3	2.1	Wnt 5A	4.4
Notch 1	2	Cadherin 5	3
		Desmocollin 3A/3B precursor	2.2
		β-catenin	2.2
		Thrombospondin 2	2.1
		FAK2	2.1
		Frizzled	2
		Desmoplakin I & II	1.8

Growth Factors & Growth Factor Receptors:

Macrophage inhibitory cytokine (MIC1)	14.2	Macrophage colony-stimulating factor 1 receptor precursor (CSF1-R, c-fms)	16.5
Heparin-binding growth factor 2 precursor (prostatropin)	11.6	IGFBP3 precursor	9.2
Low-affinity nerve growth factor receptor	4.6	Vimentin	6.5
VEGF	4.2	Fibronectin	5.5
IL-1β convertase precursor (ICE)	3.5	N-CAD (cadherin 2)	4.9
FGFR1	2.9	N-CAM	3.4
HEGR1 early growth response protein 1	2.7	Brain-derived neurotrophic factor	3.3
Fn receptor α subunit	2.6	VE-cadherin (cadherin 5)	3
Glia maturation factor β	2.2	Brain glucose transporter 3	2.9
TNFR2 (lymphotoxin β receptor)	2.2	Growth inhibitory factor (MT-III)	2.6
IL-1α precursor	2.1	Endothelin 2	2.6
TGFβ	2	Osteoclast stimulating factor	2.6
IFNα/β receptor α subunit precursor	2	Glucocorticoid receptor	2.3
IL-6 Receptor α subunit precursor	1.9	Vitronectin precursor	2.3
Mesothelin precursor (CAK-1 antigen)	1.9	Keratinocyte growth factor (FGFR2)	2.2
Myb proto-oncogene	1.9	IL-6 precursor	2.2
B-cell receptor associated protein	1.8	Glia-derived neurite promoting factor	2.2
Hepatocyte growth factor-like protein; macrophage stimulating protein	1.8	Thrombospondin 2 precursor	2.1
Oncostatin M-specific receptor β subunit	1.7	Macrophage migration inhibitory factor	2.1
		VEGFB precursor	2
		Glucocorticoid receptor α variant	2
		IFNγ Receptor	1.8
		Endothelial TNFα-induced protein	1.8
		Stem cell factor precursor (mast cell growth factor)	1.7
		EGRα	1.6
		SMAD4	1.6

Nucleic Acid Metabolism/Catabolism:

2'-5' oligoadenylate synthetase 1	12.4	5' nucleotidase precursor; ecto-nucleotidase CD73 antigen	4.4
2'-5' oligoadenylate synthetase 2	10.1	Uridine diphosphoglucose pyrophosphorylase	4.2
Endonuclease III homolog	2.6	Phosphoribosyl pyrophosphate synthetase subunit	3.7
DNA primase small subunit 49 kDA	2.6	Adenylate kinase isoenzyme 1 (ATP-AMP transphosphorylase	2.2
Uridine phosphorylase	2.1	DNA ligase IV	2.1
Adenosine de-aminase	1.9	IMP dehydrogenase I	1.9
Purine nucleoside phosphorylase (PNP) (inosine phosphorylase)	1.7	ADP/ATP carrier protein	1.7
Uridine monophosphate kinase	1.6		
Glycyl tRNA synthetase	1.6		

Extracellular Matrix/ Tissue remodeling-associated Genes:

		Serum & glucocorticoid-regulated serine-threonine protein kinase	2.9
		Growth inhibitory factor MT III	2.6
		Glucocorticoid receptor	2.3
		Cytokeratin 19	2.3
		Integrin α6 precursor	2.3
		Desmocollin 3A/3B precursor	2.2
		β-catenin	2.2
		Thrombospondin 2 precursor	2.1
		FAK2	2.1
		Macrophage migration inhibitory factor	2.1
		Glucocorticoid receptor α variant	2
		Integrin β6 precursor	2
		Integrin β5 subunit precursor	2
		Tenascin precursor	2
		Desmoplakin 1 & 2	1.8
		Collagen 4 α2 subunit precursor	1.8
		Procollagen 2 α 1 subunit precursor	1.7
		Cytokeratin 7	1.6
		Cytokeratin 2E	1.6
